# Supplementary material for: Genotype–environment interactions determine microbiota plasticity in the sea anemone Nematostella vectensis
Source: PLoS Biol. 2023 Jan 23;21(1):e3001726. doi: 10.1371/journal.pbio.3001726 (PMC9894556; doi:10.1371/journal.pbio.3001726)
Supplement: S1 Table — For Nova Scotia, Maine, and New Hampshire in March, the temperatures were inferred from previous weather reports (Reitzel and colleagues, 2013). (DOCX) [file pbio.3001726.s001.docx]

**S1 Table. Metadata and environmental data at sampling time points**. For Nova Scotia, Maine and New Hampshire in March the temperatures were inferred from previous weather reports [38].

| **Location** | **Latitude** | **Longitude** | **Collection Date** | **Temperature (C°)** | | | **Salinity (ppt)** |
| --- | --- | --- | --- | --- | --- | --- | --- |
|  |  |  |  | Avg | Max | SD |  |
| Crescent Beach, Nova Scotia | 45154713 | -64371314 | 10/03/16 | -2.20 | 1.76 | // | 28 |
| Saco, Maine | 43560682 | -70271087 | 11/03/16 | 1.95 | 5.34 | 1.93 | 24 |
| Saco, Maine | 43560682 | -70271087 | 02/06/16 | 24.63 | 27.67 | 1.98 | 23 |
| Saco, Maine | 43560682 | -70271087 | 11/09/16 | 22.48 | 25.42 | 1.92 | 28 |
| Odiorne, New Hampshire | 42964569 | -70764588 | 11/03/16 | 10.15 | 13.42 | // | 35 |
| Odiorne, New Hampshire | 42964569 | -70764588 | 02/06/16 | 22.4 | 22.4 | // | 38 |
| Odiorne, New Hampshire | 42964569 | -70764588 | 11/09/16 | 28.12 | 29.76 | // | 35 |
| Sippewissett, Massachusetts | 41577021 | -70640749 | 12/03/16 | 12.23 | 19.47 | 4.8 | 22 |
| Sippewissett, Massachusetts | 41577021 | -70640749 | 03/06/16 | 21.71 | 26.2 | 2.81 | 24 |
| Sippewissett, Massachusetts | 41577021 | -70640749 | 13/09/16 | 22.63 | 33.26 | 5.67 | 25 |
| Ft. Fisher, North Carolina | 34487519 | -77439831 | 16/03/16 | 22.42 | 31.98 | 5.22 | 28 |
