# Supplementary material for: Genotype–environment interactions determine microbiota plasticity in the sea anemone Nematostella vectensis
Source: PLoS Biol. 2023 Jan 23;21(1):e3001726. doi: 10.1371/journal.pbio.3001726 (PMC9894556; doi:10.1371/journal.pbio.3001726)
Supplement: S2 Table — NS (Nova Scotia), ME (Maine), NH (New Hampshire), MA (Massachusetts), MD (Maryland), NC (North Carolina), numbers near the location abbreviations indicate the different genotypes; colors represent the different significance (yellow = p ≤ 0.05, blue = p ≤ 0.01, green = p < 0.001). (DOCX) [file pbio.3001726.s002.docx]

**S2 Table. Dunn’s post-hoc comparisons of the Kruskal-Wallis test performed on data represented in Fig. 5F.** NS (Nova Scotia), ME (Maine), NH (New Hampshire), MA (Massachusetts), MD (Maryland), NC (North Carolina), numbers near the location abbreviations indicate the different genotypes; colors represent the different significance (yellow = p ≤ 0.05, blue = p ≤ 0.01, green = p < 0.001).

******
