# Supplementary material for: Genotype–environment interactions determine microbiota plasticity in the sea anemone Nematostella vectensis
Source: PLoS Biol. 2023 Jan 23;21(1):e3001726. doi: 10.1371/journal.pbio.3001726 (PMC9894556; doi:10.1371/journal.pbio.3001726)
Supplement: S1 Fig — (Jaccard metric, sampling depth = 5,000). Underlying data can be found in S1 Data. (DOCX) [file pbio.3001726.s005.docx]

**
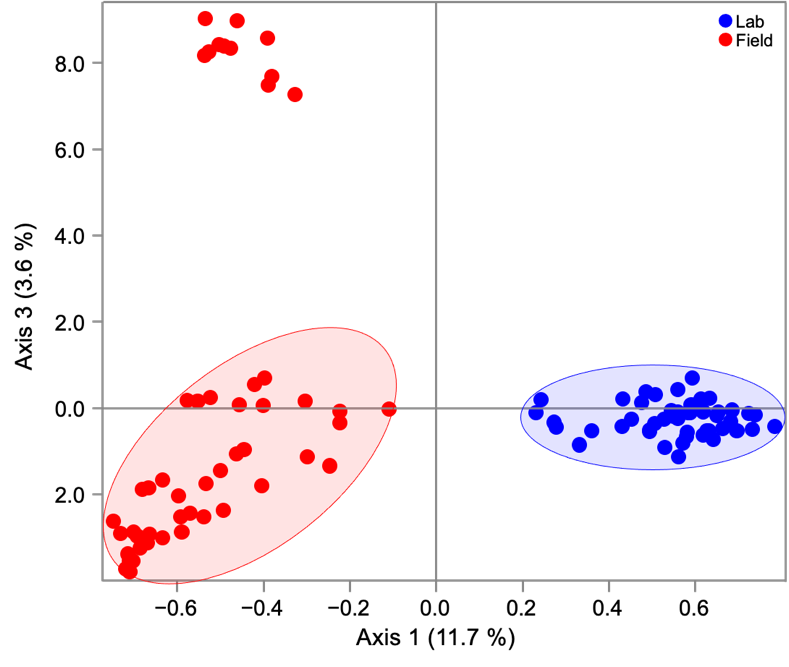
**

**S1 Fig. PCoA illustrating similarity of bacterial communities based on sample source (**Jaccard metric, sampling depth = 5000). Underlying data can be found in S1 Data.
