# Supplementary material for: Genotype–environment interactions determine microbiota plasticity in the sea anemone Nematostella vectensis
Source: PLoS Biol. 2023 Jan 23;21(1):e3001726. doi: 10.1371/journal.pbio.3001726 (PMC9894556; doi:10.1371/journal.pbio.3001726)
Supplement: S2 Fig — Underlying data can be found in S1 Data. (DOCX) [file pbio.3001726.s006.docx]

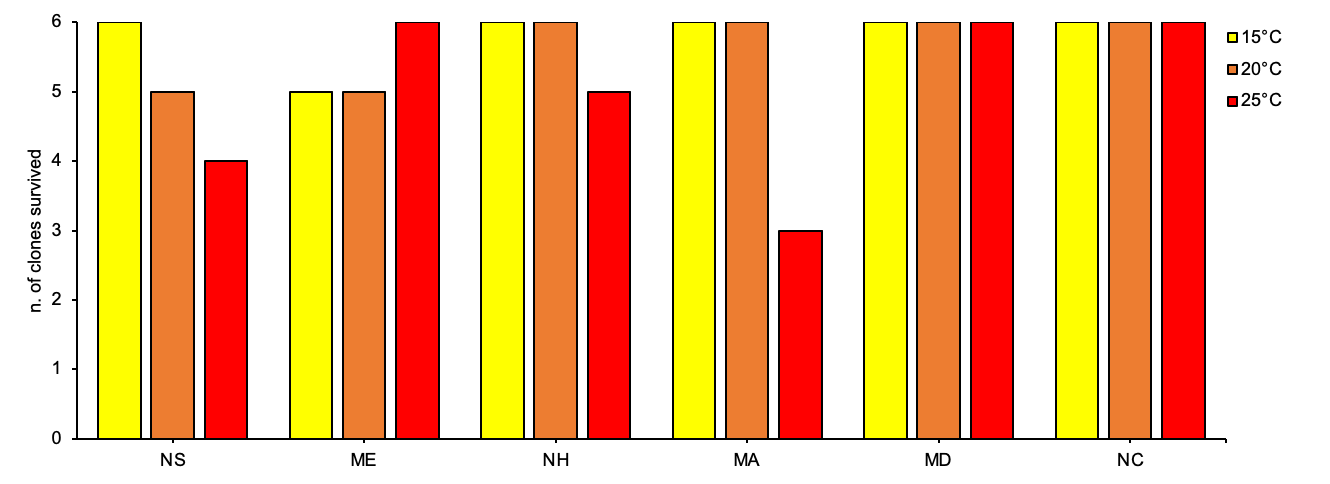


**S2 Fig. Number of clones for each provenance location that survived at the 3 different temperatures.** Underlying data can be found in S1 Data.
