# Supplementary material for: Genotype–environment interactions determine microbiota plasticity in the sea anemone Nematostella vectensis
Source: PLoS Biol. 2023 Jan 23;21(1):e3001726. doi: 10.1371/journal.pbio.3001726 (PMC9894556; doi:10.1371/journal.pbio.3001726)
Supplement: S3 Fig — (A) Beta-diversity distance box plots between different temperatures (Jaccard metric, sampling depth = 15,800); (B) alpha-diversity comparisons between temperatures (max rarefaction depth = 15,800, num. steps = 10). Differences were tested through Kruskal–Wallis test (not significant). Underlying data can be found in S1 Data. (DOCX) [file pbio.3001726.s007.docx]

***
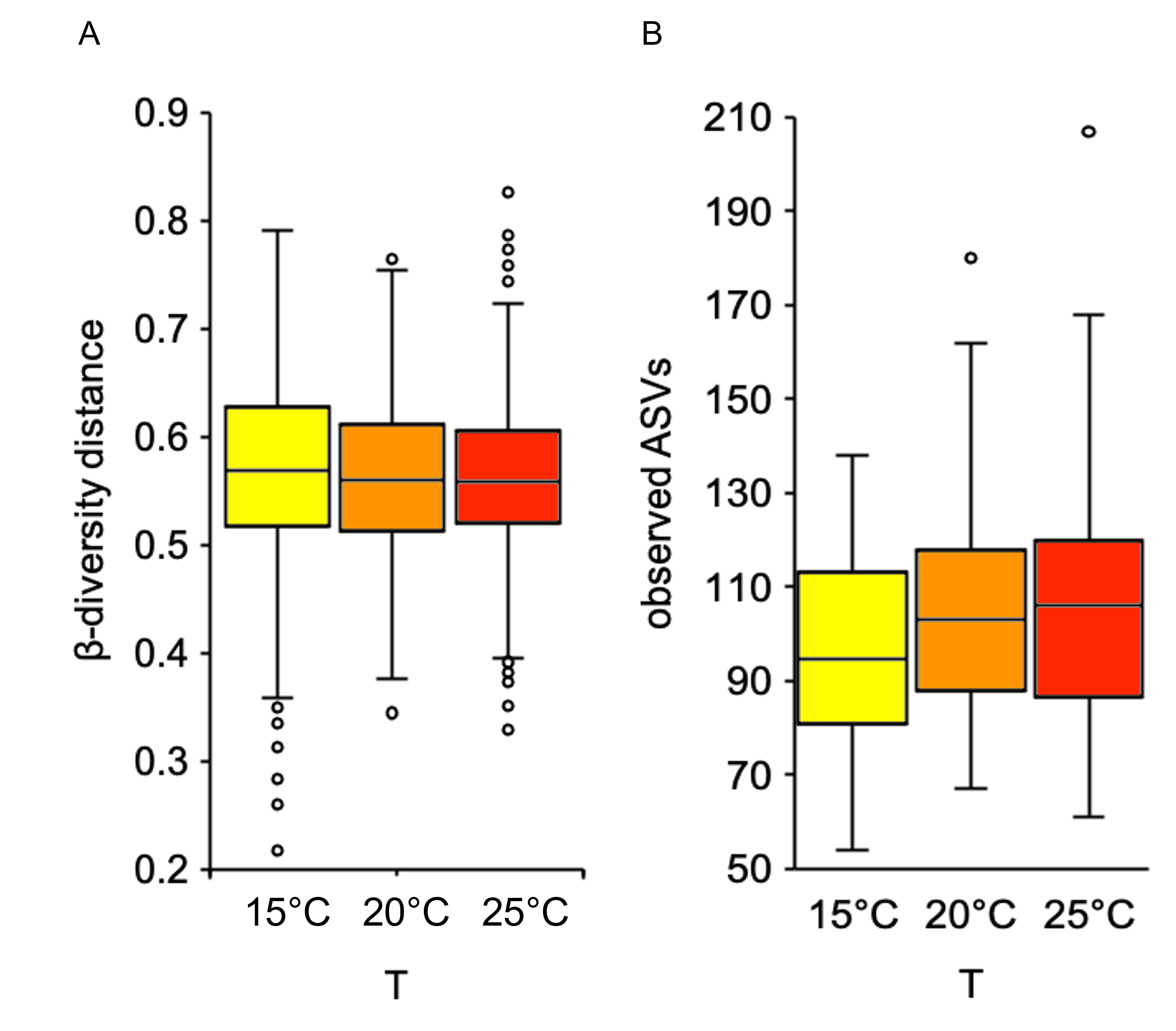
***

**S3 Fig.** **Beta-diversity and alpha-diversity distance comparisons between temperatures.** A) Beta-diversity distance box-plots between different temperatures (Jaccard metric, sampling depth = 15800). B) Alpha-diversity comparisons between temperatures (max rarefaction depth = 15800, num. steps = 10). Differences were tested through Kruskal-Wallis test (not significant). Underlying data can be found in S1 Data.
