# Supplementary material for: Genotype–environment interactions determine microbiota plasticity in the sea anemone Nematostella vectensis
Source: PLoS Biol. 2023 Jan 23;21(1):e3001726. doi: 10.1371/journal.pbio.3001726 (PMC9894556; doi:10.1371/journal.pbio.3001726)
Supplement: S4 Fig — (max rarefaction depth = 15,800, num. steps = 10) (Jaccard metric, sampling depth = 15,800), differences were tested through Kruskal–Wallis test (H = 38.91, p = < 0.001); for clarity the Dunn’s post hoc comparisons are reported in the table. NS (Nova Scotia), ME (Maine), NH (New Hampshire), MA (Massachusetts), MD (Maryland), NC (North Carolina), numbers near the location abbreviations indicate the different genotypes. Underlying data can be found in S1 Data. (DOCX) [file pbio.3001726.s008.docx]

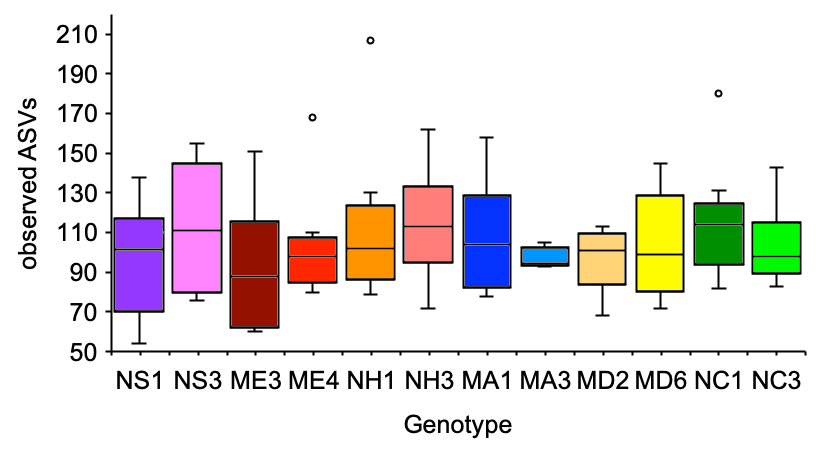


**S4 Fig.** **Alpha-diversity comparisons between polyp genotypes** (max rarefaction depth = 15800, num. steps = 10) (Jaccard metric, sampling depth = 15800), differences were tested through Kruskal-Wallis test (H = 38.91, p = < 0.001); for clarity the Dunn’s post-hoc comparisons are reported in the table. NS (Nova Scotia), ME (Maine), NH (New Hampshire), MA (Massachusetts), MD (Maryland), NC (North Carolina), numbers near the location abbreviations indicate the different genotypes. Underlying data can be found in S1 Data.
