# Supplementary material for: Genotype–environment interactions determine microbiota plasticity in the sea anemone Nematostella vectensis
Source: PLoS Biol. 2023 Jan 23;21(1):e3001726. doi: 10.1371/journal.pbio.3001726 (PMC9894556; doi:10.1371/journal.pbio.3001726)
Supplement: S5 Fig — NS (Nova Scotia), ME (Maine), NH (New Hampshire), MA (Massachusetts), MD (Maryland), NC (North Carolina), numbers near the location abbreviations indicate the different genotypes. Underlying data can be found in S1 Data. (DOCX) [file pbio.3001726.s009.docx]

*
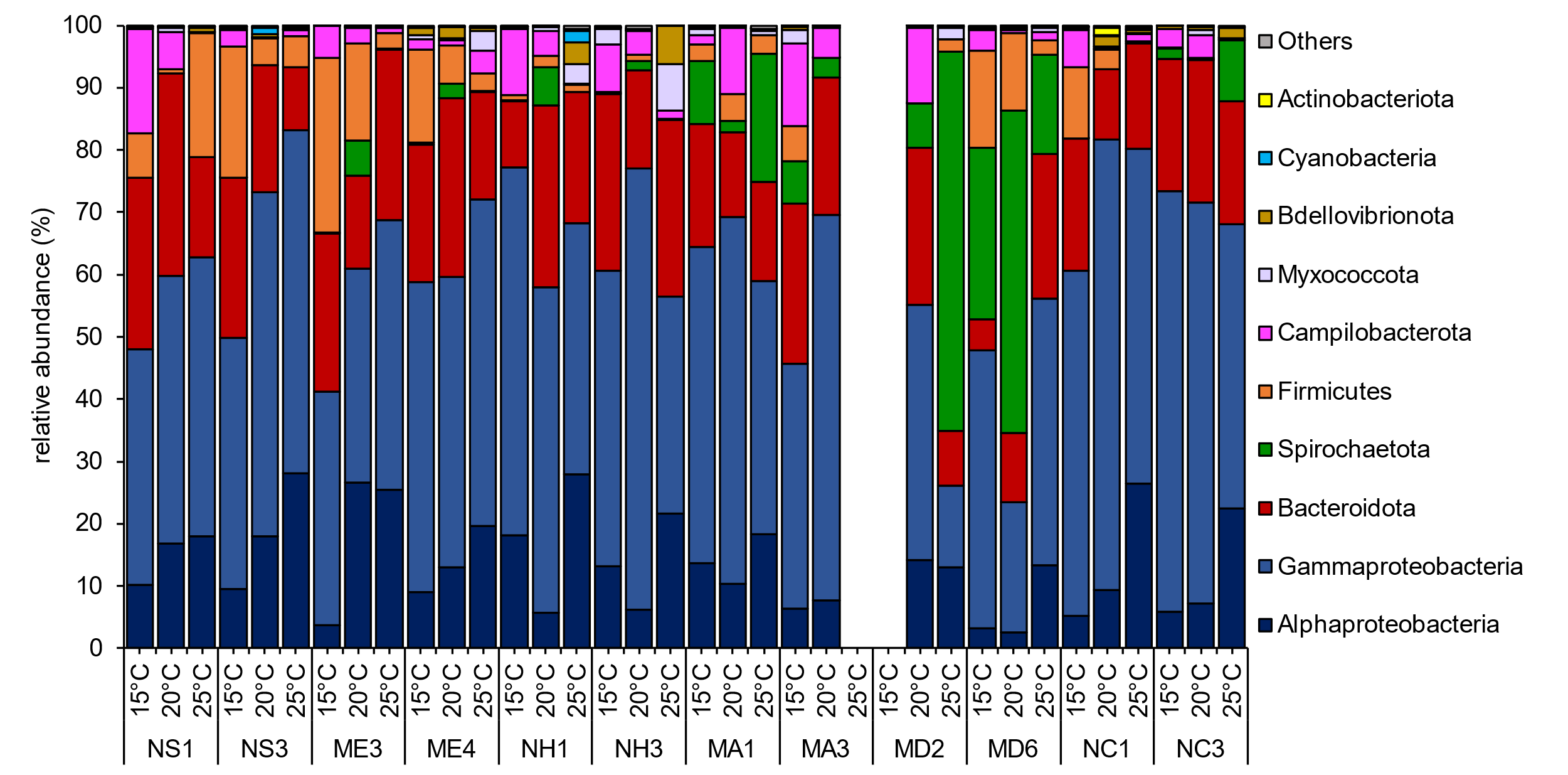
*

**S5 Fig. Relative abundance of main bacterial groups among the different genotypes at the three temperatures.** NS (Nova Scotia), ME (Maine), NH (New Hampshire), MA (Massachusetts), MD (Maryland), NC (North Carolina), numbers near the location abbreviations indicate the different genotypes. Underlying data can be found in S1 Data.
