# Supplementary material for: Genotype–environment interactions determine microbiota plasticity in the sea anemone Nematostella vectensis
Source: PLoS Biol. 2023 Jan 23;21(1):e3001726. doi: 10.1371/journal.pbio.3001726 (PMC9894556; doi:10.1371/journal.pbio.3001726)
Supplement: S6 Fig — A and B show the same samples of Fig 5E divided by similar reaction norms. NS (Nova Scotia), ME (Maine), NH (New Hampshire), MA (Massachusetts), MD (Maryland), NC (North Carolina), numbers near the location abbreviations indicate the different genotypes. Underlying data can be found in S1 Data. (DOCX) [file pbio.3001726.s010.docx]

*
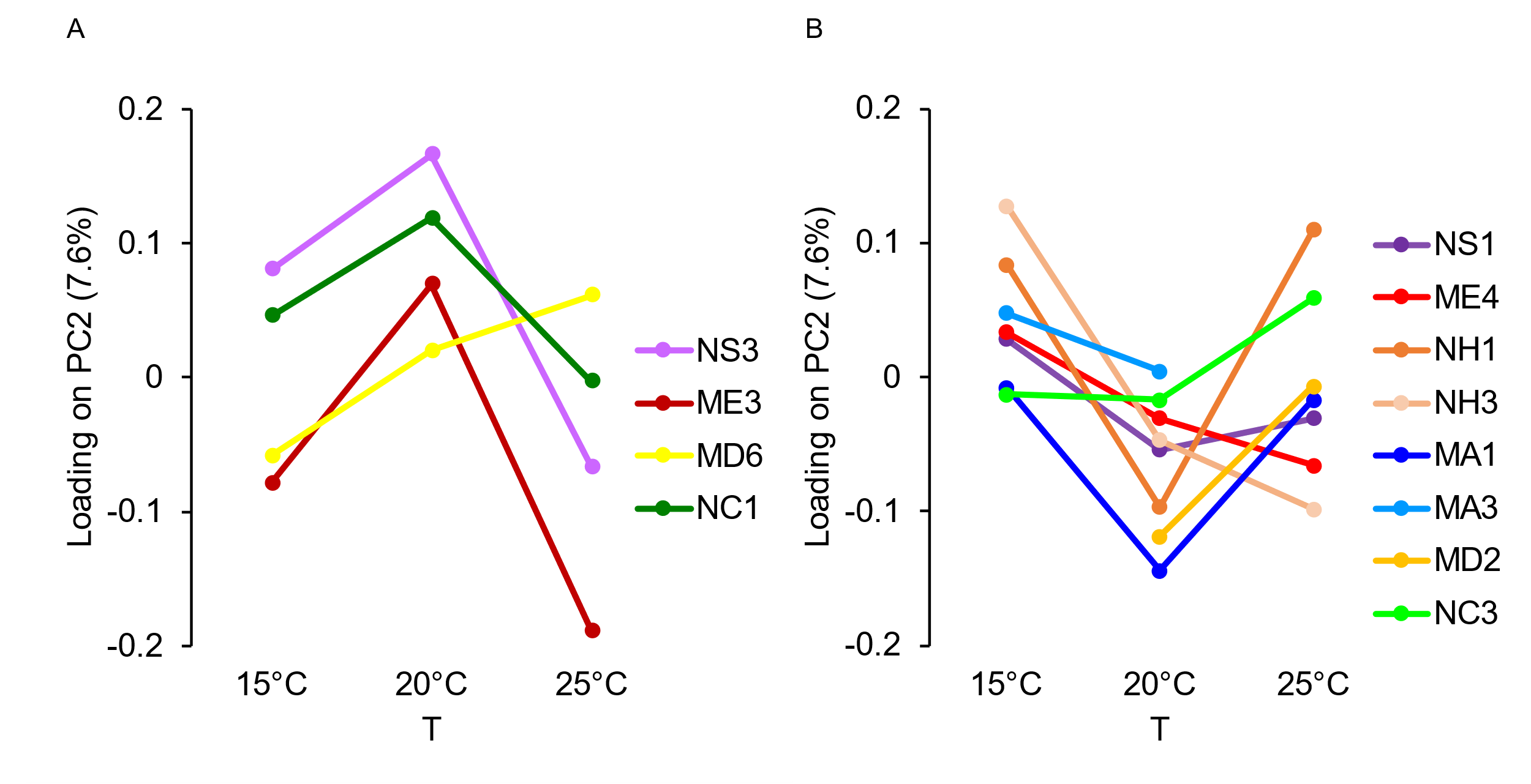
*

**S6 Fig. Reaction norms.** A and B show the same samples of Fig. 5G divided by similar reaction norms. NS (Nova Scotia), ME (Maine), NH (New Hampshire), MA (Massachusetts), MD (Maryland), NC (North Carolina), numbers near the location abbreviations indicate the different genotypes. Underlying data can be found in S1 Data.
