# Supplementary material for: Genotype–environment interactions determine microbiota plasticity in the sea anemone Nematostella vectensis
Source: PLoS Biol. 2023 Jan 23;21(1):e3001726. doi: 10.1371/journal.pbio.3001726 (PMC9894556; doi:10.1371/journal.pbio.3001726)
Supplement: Attachment — Submitted filename: Response to reviewer.docx [file pbio.3001726.s012.docx]

We thank all the reviewers for their suggestions and contributions, that we believe helped us to improve the consistency and impact of our story.

**Reviewer #1:**

Overall comments: I think there could be an interesting story here, but there are some major issues that need to be addressed first:

1. The data analysis needs a refresh. This paper is ALL about the *N. vectensis* microbiota but it was analysed with a tool from 2016-17. So much has changed (for the better) in the last five years. I recognize this is a time-consuming process, but this is the only way that the MS can achieve as high of an impact as PLOS Biology. I would also step away from the OTU approach and look at ASVs (or at least bin OTUs to a greater identity). No stats should be taken from QIIME2 as it does not allow the user to add in random effects.

**Answer:** We re-analysed the whole dataset using Qiime 2. The two pipelines return consistent and comparable results. This was already shown in other studies (García-López et al., 2021; Glassman & Martiny, 2018; Joos et al., 2020; Moossavi et al., 2020; Porter & Hajibabaei, 2020). Therefore, our general conclusions are maintained using ASVs instead of OTUs (97%), while the annotation of reads differ significantly, due to an updated reference database. To be able to add random effects to our statistically analyses, we used Primer7 for downstream analyses.

2. Genotype becomes a factor in the second experiment; however,

a. There is no description of the way that the authors determined that animals collected from a same (or different site) were unique genotypes.

Answer: We propagated individually sampled polyps asexually in the lab resulting in clonal cultures. We selected from each population two clonal cultures for the acclimation experiment. In previous studies, which were performed on similar natural populations of *N. vectensis*, (Darling et al., 2004; Reitzel et al., 2008, 2010) it was shown that individuals from each population are genetically different with significant genetic separations of alleles/genotypes (e.g., F_ST_, phylogenetic relationships).

b. There were only two genotypes per site. The authors show that geographic site had a significant impact on microbiota composition (see Fig. 2B). The only "genotypic" differences were between animals from different sites (i.e., Fig. 5F - this is hardly a genotype effect. c. Statistically speaking, genotype effects cannot be addressed without considering collection site.

**Answer:** To proof that we observed a genotype effect, we included the geographic site (“location”) as random factor into our analyses. The significance is maintained for the genotype, as well as for the genotype*temperature effect. The presence of different genotype within one population is also supported by the fact, that genotypes from the same geographic site show significant differences in their microbiota plasticity (Figure S6) and microbiota flexibility (Figure 5D, Table S2), which can only be explained by host genetics.

3. Some experimental decisions need an explanation.

a. The decision to transfer collected animals from their environmental conditions (ranging -2.2 to 22.4 C according to Table S1) and transfer them to 20C.

**Answer:** To maintain field collected animals in the laboratory, animals were acclimated to the standard laboratory conditions to facilitate easy maintenance.

b. Using two different extraction kits and not including (or presenting) experimental controls in the sequencing run.

**Answer:** We agree with the reviewer's comment that using two different DNA extraction kits was not ideal. However, the work was divided into two parts. The first part consists of a comparison of animals taken from the field and those kept under laboratory conditions for one month. These samples were extracted directly in the USA using the AllPrep DNA/RNA Mini Kit (Qiagen). The second part consists of experimental acclimation for three months at three different temperatures. This experiment was performed in Germany and samples were extracted using the DNeasy Blood & Tissue Kit (Qiagen). Since we analyzed both data sets separately and did not make any intercomparisons, we avoided conclusions based on possible extraction differences.

4. Discussion needs to be flushed out and address the following topics in more detail:

a. Core microbiome. Which microbiota are essential? How consistent is this? What could they be doing?

**Answer:** We disagree with the reviewer on the point that a detailed consideration of the "core microbiota" would strengthen our story. We agree that uncovering the function of bacterial colonizers is a key question for the future. Indeed, we are currently addressing these questions in our laboratory and have recently published a first manuscript on this point. However, bacterial function goes far beyond the focus of this manuscript and cannot be revealed by a "core microbiota" analysis. Furthermore, we argue that the flexible part of the microbiota is most likely as important as the constant part (core microbiota), as shown in Baldassarre et al, 2022.

b. More of a literature review required on the changes in cnidarian microbial community structure when moved from field to laboratory conditions. There are several papers available.

**Answer:** We agree with the reviewer and added more references addressing this point.

Line specific comments:

L132: Are you saying that all only March animals were exposed to lab conditions? What are "standard lab conditions"? Maybe site a paper or say "refer below." The sampling text needs a little more clarity or perhaps a small figure. Its an important part of the story to know what samples are from the field vs lab, and then which animals were exposed to the temperature stress.

**Answer:** Yes, only animals sampled in March were divided into two parts. In addition, we clarified laboratory conditions and cited the original paper: “Half of the animals from March sampling were kept for one month in the laboratory, under constant, artificial conditions, at 20°C, without substrate or light, in *N. vectensis* Medium (NM), which was adjusted to 16ppt salinity with Red Sea Salt and Millipore H_2_O (according to (Hand & Uhlinger, 1992).”

L134: Ok - so you collected animals at a variety of temperatures (ranging -2.2 to 22.4 C according to Table S1) but then held them all in lab conditions at 20C? How did you ramp that? How did you consider this stress in your future experiments? How do you determine that 1 month was enough to be acclimated?

**Answer**: We did not ramp the temperature for the acclimation of the animals to the lab conditions. We have routinely cultured *N. vectensis* in the lab following field collection using this approach without any mortality or clear indications of stress. Individuals routinely display behaviours indicating they are unstressed (e.g., full body extension, feeding). We did not conduct any preliminary experiments to determine if 1 month is sufficient time for acclimation; however, the acclimation time we used exceeds previous research with *N. vectensis* that used acclimation times of ~2 weeks for experiments comparing field and lab cultured anemones (Tarrant et al., 2019).

L135: I would prefer a psu or ppt value here instead.

**Answer**: We changed it accordingly.

L142: How do you know they were two different strains?

**Answer:** We propagated individually sampled polyps asexually in the lab resulting in clonal cultures. We selected from each population two clonal cultures for the acclimation experiment. In previous studies, which were performed on similar natural populations of *N. vectensis*, (Darling et al., 2004; Reitzel et al., 2008, 2010) it was shown that individuals from each population are genetically different with significant differences in alleles/genotypes.

We added the following information to the text (L149): “individually sampled polyps from six wild populations were asexually propagated for more than one year under laboratory conditions. After that, two strains from each original population were selected for the following experiment. Three polyps (three replicates) for each of the twelve strains selected were placed separately into 6 well plates and let acclimate for three months at each of the three different acclimation temperatures (15, 20 and 25°C)…..”

L148: Not great that you used two different kits for gDNA extraction - especially when you want to compare the samples. Worthwhile to mention this possible point of difference in the discussion (or perhaps you'll find a paper that has tested the differences between these two kits).

**Answer:** We agree with the reviewer's comment that using two different DNA extraction kits was not ideal. However, the work was divided into two parts. The first part consists of a comparison of animals taken from the field and those kept under laboratory conditions for one month. These samples were extracted directly in the USA using the AllPrep DNA/RNA Mini Kit (Qiagen). The second part consists of experimental acclimation for three months at three different temperatures. This experiment was performed in Germany and samples were extracted using the DNeasy Blood & Tissue Kit (Qiagen). Since we analyzed both data sets separately and did not make any intercomparisons, we avoided conclusions based on possible extraction differences.

L159: I don't see any mention of controls - seawater controls, Artemia feed, or at least extraction blanks. Especially because you used two different kits.

**Answer:** Blank tests were performed in all of our experiments. We have included this information in the M&M section. However, we did not perform *Artemia* or seawater controls. We are not convinced of the informative value of these controls. It is probably true that both factors affect the microbiota of *N. vectensis*, however, in the laboratory experiments, all animals were fed the same *Artemia*. Therefore, we can conclude that the observed differences in the microbiota composition cannot be due to the *Artemia* themselves. Also, in the field samples, the aquatic microbiota most likely infiltrates the microbiota of *N. vectensis*. But there are other environmental microbiota that are likely similarly important, such as the sediment microbiota and the microbiota of potential prey organisms. All of these factors accumulated in the tested effect of sampling location. Because we were interested in the abiotic effects in our experiments, we did not analyse the environmental microbiota in details.

L182: The data was processed with QIIME (v1)? What reference database? It's also not idea that you used OTUs. I would suggest rerunning in QIIME2 (we're currently up to v2022.2) as your data was processed in a version older than 2017 and A LOT has changed when it comes to metabarcoding in the last 5 years. While I don't object to the OTU model, amplicons should be binned at a higher resolution (i.e. 99% identity) or just use ASV.

**Answer:** As requested, we reprocessed the whole dataset through Qiime2 against the Silva database (last available versions) and used 100% identity ASVs.

L194: The statistical capability of QIIME2 (which is confusing that you used this when you analyzed your data in QIIME1) is very limited. There is no capacity to add random effects among other things. The data needs to be exported and analysed in external software - R would be great.

**Answer:** We reanalysed the whole dataset with Qiime2 and performed downstream statistical tests with Primer7, which also allows to test for random effect.

L325: The title of figure 4 is "Population-specific microbiota are maintained under laboratory conditions," except no part of this figure compares a given geographic region over time in laboratory conditions.

**Answer:** All samples shown in Figs. 2 and 4 are from animals sampled in March in the field. We show that the clustering according to geographic location observed in animals sampled in the wild is maintained and even enhanced after one month of laboratory culture.

L339: There is nothing in the methods to explain how you knew they were distinct genotypes. This needs to be explained.

**Answer:** We selected two clonal cultures from each population for the acclimation experiment. In previous studies conducted on similar natural populations of *N. vectensis* (Darling et al., 2004; Reitzel et al., 2008, 2010), it has been shown that population differ greatly in their genetics. Even individuals from the same population were genetically different, as the occurrence of clones in natural populations is very rare. (Darling et al. 2004, Reitzel et al. 2008).

L370: This figure should be called Figure 5. I like Fig. 5A - could you have some version of that above to help explain the sampling system? Perhaps add to 5A that these were all collected in March, so the same as time point 1. Something else I don't quite understand - since these animals were collected from a range of geographic sites, did each of their "ambient" temperatures correspond to collection temperatures? I think you could get rid of Figures 5C-G. They don't add to your story and the data you present here can be sufficiently provided as text.

**Answer:** We agree, this should be Figure 5. The twelve genotypes for the acclimation experiments were not all sampled in March 2016. However, all genotypes were long-term cultured (>1 year) at laboratory conditions, before they were used for the experiment. The two experiments (field sampling and acclimation experiment) are separated from each other. The commonality of these two experiments is that the animals originate from the same populations. We clarified this in the method section. The temperature range of 15 to 25 °C used for the acclimation experiment is within the tolerance range of N. vectensis, is a good permissive temperatures commonly encountered in their native habitats, and were used in previous studies (Baldassarre et al., 2022; Reitzel et al., 2013).

In part, we agree with your opinion that Figures 5C-G are less important. Therefore, we have moved Figure 5C and D to the Supplement. Figure 5F (now 5D) has been replaced with its counterpart from the Supplement because we found interesting differences in beta diversity distances among genotypes based on the new analyses.

We added the following paragraph to the manuscript (L337): “Interestingly, comparison of the beta diversity distances of the different genotypes (Figure 5D) revealed that they differ significantly (Kruskal Wallis p < 0.001) in terms of microbiota flexibility (Table S2). These results suggest that each genotype is endowed with a microbiota that exhibits genotype-specific flexibility. In particular, we identified genotypes whose microbiota exhibit low flexibility (e.g., MA1 and MD2), in contrast to genotypes whose microbiota exhibit high flexibility (e.g., NS3 and NH3).”

Figure 5E and G (now 5G and E) we decided to keep in the main text.

L411: There are also plenty of cnidarian studies that show reductions in microbiota diversity from the field to the lab. This one comes to mind: Damjanovic, K., et al. (2020). "Assessment of bacterial community composition within and among Acropora loripes colonies in the wild and in captivity." Coral Reefs 39(5): 1245-1255. We also know that we can reduce the diversity of Exaiptasia in the lab by just changing its environmental conditions: Dungan, A. M., et al. (2021). "Short-Term Exposure to Sterile Seawater Reduces Bacterial Community Diversity in the Sea Anemone, Exaiptasia diaphana." Front Mar Sci 7.

**Answer:** We thank the reviewer for the suggestions. We added the references into the text.

L416: I think you'll find, this really isn't all that surprising. Cnidarians eat a lot of diverse things in the wild, including bacteria (lots of papers here you can cite). When we bring them into the lab we restrict their diet. You can't go back in time to change your experiment, but I would suggest mentioning ways that you could track where these changes (i.e., reduced diversity come from), such as by taking experimental seawater samples, sequencing the Artemia you're feeding your animals, and using approaches that look at the viable microbiota instead of traditional PCR which captures dead bacteria as well as eDNA.

**Answer:** We agree with the reviewer that the phenomenon of reduced microbiota diversity in laboratory-maintained animals is not new and has been well described for many organisms. Therefore, the focus of our study was not to analyse the factors responsible for changes in the composition of the microbiota. Furthermore, we are not convinced that sequencing the seawater and *Artemia* microbiota would help us understand the causes of diversity loss. We would merely add another layer for further correlation. We aimed to show that the microbiota still retains a fingerprint of the biogeography of its host despite the enormous impact that laboratory conditions have on the physiology of animals and their symbionts. We agree that using 16S rRNA sequencing based on RNA might reduce the environmental signal in the microbiota samples. We added the following sentence to the discussion (L400): “In future studies, the amount of bacterial sequences derived from dead bacteria or eDNA could be reduced by sequencing bacterial RNA instead of DNA.”

L419: Yep, you're on the right track. Please add some citations here.

**Answer:** We added two citations here.

L421: "Bacteria that are persisting during laboratory maintenance most likely represent bacteria that are functionally associated with *N. vectensis* and might have co-evolved with its host." I think this is a really interesting idea and would like to see the discussion expanded as such. You could look for "core microbiome members" with the data set you have.

**Answer:** We agree with the reviewer that this is an interesting idea. But revealing the function of bacterial colonizers is beyond the focus of this manuscript and cannot be revealed by a "core microbiota" analysis. However, to expand on this point, we have added our recent work on bacterial function during thermal acclimation in *N. vectensis*.

L430: You mention the Motzfeld et al., 2016 paper several times but fail to compare your data to theirs. What is similar? This goes to my comment above about looking for core microbiome members. You could be sitting on a gold mine.

**Answer:** We do not believe that a comparison of the two data sets could be informative. The sampling in the Mortzfeld et al. paper was based on a much smaller sample size, and the bacterial 16S rRNA genes were sequenced using 454 sequencing with very low coverage. For this reason, we expanded the data set by sampling these populations on a broader scale to derive common signatures beyond those in the Mortzfeld et al. paper. We do not expect to gain any further insights from comparing the two datasets.

L457: You really don't have the dataset to call a section "Microbial plasticity is promoting animal adaptations." You didn't measure any physiology and it would appear that you actually stressed out some animals by shifting them to 20C.

**Answer:** We agree with the reviewer that this heading is not based solely on this manuscript, but on a combination of the results presented here and earlier work cited in this paragraph. That is the purpose of a discussion. We therefore change the heading only slightly to: "Microbial plasticity is linked to animal adaptations".

**Reviewer #2:**

This manuscript a) describes natural variation across biogeography in the microbial associates of an anemone b) with regard additionally to season and c) clone identity and d) thermal environment (experimental in the laboratory). The analyses are 'broad brush' in that they are based on 16S rRNA sequence, which is a very broad classification tool, and indicate changes in relative abundance, rather than actual abundance. In general the depth of analyses and experiments were strong and patterns were, to me, clear. There is natural latitudinal variation, clone variation, and thermal impacts.

What is less clear is the broader significance of the findings of this paper. Neither the abstract, introduction nor the discussion gave a compelling case, to me, of why this manuscript reaches beyond a more specialist audience. The microbe changes are not dissected functionally - they are groups of bacteria that change, but how these bacteria live and their metabolism and potential interaction with hosts is not dissected - they remain relatively anonymous taxonomic bins without supporting genomics that might highlighted their biological significance. Functional significance is mentioned only in the discussion in reference to a previously (2021) published preprint. That preprint paper I can see the broader significance of, as it demonstrates a phenotypic functional consequence of the plasticity observed (very interesting); I found this paper more descriptive, essentially reporting clone, thermal and clone x thermal effects on microbiota which are clear, but are to me relatively expected and well executed but routine findings (as is the simplification of microbiota in lab culture, which is commonly seen).

**Answer:** We thank the reviewer for the comment. In our manuscript, we show compelling evidence that plasticity of the microbiota, which is an important determinant of local thermal acclimation and adaptation (see Baldassarre et al. 2022), is genotype specific. We believe that this result is of general interest to a broader readership because, to our knowledge, this interaction has not been previously demonstrated in other organisms. Future experiments will reveal how this genotype-specific plasticity affects the ability to cope with changing environmental conditions.

**Reviewer #3:**

The manuscript attempts to address a fundamental question regarding the determinants of microbes associated with animals. This is an essential question because microbes can significantly affect animal physiology, including disease. The research conducts excellent experiments merging natural collections and lab-rearing experiments in *Nematostella* to tease apart genotype and environmental contributions to the microbiome. In general, I think this work sets the stage for crucial functional analysis of the role of microbes in *Nematostella* physiology. The experimental design is beautiful, and I hope the authors take the next step and try to discover the roles of particular microbes.

I have three major questions about the work:

First, it is unclear which animals from which collections were used in each analysis. It may be eluded to or obvious to the writer, but I was counting dots on the plots to determine which animals were included in each figure. This is an essential aspect of the experimental design to clarify. I suggest explicitly adding this information to the figures and results text. For example, if all wild animals were used in the analysis in Fig. 2, genotype may co-vary with collection time. These co-variates may also be addressed through statistical analysis. However, subsetting the data for each comparison is also fair.

**Answer:** The reviewer is right, that we have subsetted the analyses and the animals shown in Figure 2 are only those sampled in March 2016. All animals sampled in the other months were removed from this analysis. We added this information to the text and to the corresponding figure legend. In Figure 3 all field sampled polyps were included to have a more consistent comparison with the lab samples; we clarified this a better in the text.

Second, after reading the paper, I was excited to read the end of the discussion (lines 457-471) and the article by Baldassarre 2021 (citation needs updates). I found this work fascinating and directly related to the current study. However, I think the Baldassarre paper needs to be introduced in the introduction and the knowledge gaps highlighted. Then, the results of the present study should be placed in the context of the Baldassarre paper. This would help the non-expert understand how these two studies relate, overlap, and how the field is progressing.

**Answer:** We thank the reviewer for the comment. We have added the following paragraph to the introduction (L103) to better link the two studies: “Recently, we have shown that genetically identical animals differentiate their microbiota composition in response to a change in environmental temperature. By transplanting the adapted microbiota onto non-adapted animals, we demonstrated that the observed microbiota plasticity leads to increased tolerance of the animals to thermal stress (Baldassarre et al., 2022). However, the influence of the host genotype on the plasticity of the microbiota and thus on the ability to cope with changing environmental conditions remained unclear.”

Third, the data and results are precise. However, the manuscript might benefit from discussing the importance or functional consequences of these shifts in microbes. Are there clear hypotheses generated by these data that may be testable in future studies? What are the major outstanding questions? This will help the reader see the data in a broader context and may inspire the field.

**Answer:** We agree with the reviewer and added the following paragraph addressing new hypothesis generated by our results and potential future studies (L 467): “Together these results may indicate that the genotype-specific bacterial colonization reflects local adaptation. Future studies will reveal whether lower plasticity and flexibility of the microbiota is associated with lower adaptability to changing environmental conditions and which host factors determine the plasticity and flexibility of the microbiota. In particular, genotypes adapted to highly variable environments might favour flexibility over fidelity regarding the associated microbiota composition; conversely, under more stable conditions less dynamic and more strict association might be advantageous (Voolstra & Ziegler, 2020).

Minor points:

Lines 32-35: Not really true that five populations of *Nematostella* were isolated three times during the year. It was only a subset of populations. Minor point, but you may consider clarifying.

**Answer:** The reviewer is right, that only one population was sampled three times in the year. We corrected the sentence accordingly.

There are two figures 1 and 2. I think they should be 4 and 5.

**Answer:** The reviewer is right. The last figures should be Figure 5 and 6. We corrected this in the revised manuscript.

Figure 2: It is impossible to tell the differences between many of the colors in this plot. Also, the key is not readable.

**Answer:** The different colors in the diagram in Figure 2 indicate the different sampling locations. We have added this information to the legend of the figure. We increased the keys in Figure 2 B and E to make them readable.

**References**

Baldassarre, L., Ying, H., Reitzel, A. M., Franzenburg, S., & Fraune, S. (2022). Microbiota mediated plasticity promotes thermal adaptation in the sea anemone Nematostella vectensis. *Nature Communications*, *13*(1), 3804. https://doi.org/10.1038/s41467-022-31350-z

Darling, J. A., Reitzel, A. M., & Finnerty, J. R. (2004). Regional population structure of a widely introduced estuarine invertebrate: Nematostella vectensis Stephenson in New England. *Molecular Ecology*, *13*(10), 2969–2981. https://doi.org/10.1111/j.1365-294X.2004.02313.x

García-López, R., Cornejo-Granados, F., Lopez-Zavala, A. A., Cota-Huízar, A., Sotelo-Mundo, R. R., Gómez-Gil, B., & Ochoa-Leyva, A. (2021). OTUs and ASVs Produce Comparable Taxonomic and Diversity from Shrimp Microbiota 16S Profiles Using Tailored Abundance Filters. *Genes*, *12*(4), Art. 4. https://doi.org/10.3390/genes12040564

Glassman, S. I., & Martiny, J. B. H. (2018). Broadscale Ecological Patterns Are Robust to Use of Exact Sequence Variants versus Operational Taxonomic Units. *mSphere*, *3*(4), e00148-18. https://doi.org/10.1128/mSphere.00148-18

Hand, C., & Uhlinger, K. R. (1992). The culture, sexual and asexual reproduction, and growth of the sea anemone Nematostella vectensis. *Biological Bulletin*, *182*(2), 169–176. https://doi.org/10.2307/1542110

Joos, L., Beirinckx, S., Haegeman, A., Debode, J., Vandecasteele, B., Baeyen, S., Goormachtig, S., Clement, L., & De Tender, C. (2020). Daring to be differential: Metabarcoding analysis of soil and plant-related microbial communities using amplicon sequence variants and operational taxonomical units. *BMC Genomics*, *21*(1), 733. https://doi.org/10.1186/s12864-020-07126-4

Moossavi, S., Atakora, F., Fehr, K., & Khafipour, E. (2020). Biological observations in microbiota analysis are robust to the choice of 16S rRNA gene sequencing processing algorithm: Case study on human milk microbiota. *BMC Microbiology*, *20*(1), 290. https://doi.org/10.1186/s12866-020-01949-7

Porter, T. M., & Hajibabaei, M. (2020). Putting COI Metabarcoding in Context: The Utility of Exact Sequence Variants (ESVs) in Biodiversity Analysis. *Frontiers in Ecology and Evolution*, *8*, 248. https://doi.org/10.3389/fevo.2020.00248

Reitzel, A. M., Chu, T., Edquist, S., Genovese, C., Church, C., Tarrant, A. M., & Finnerty, J. R. (2013). Physiological and developmental responses to temperature by the sea anemone Nematostella vectensis. *Marine Ecology Progress Series*, *484*(Somero 2012), 115–130. https://doi.org/10.3354/meps10281

Reitzel, A. M., Darling, J. A., Sullivan, J. C., & Finnerty, J. R. (2008). Global population genetic structure of the starlet anemone Nematostella vectensis: Multiple introductions and implications for conservation policy. *Biological Invasions*, *10*(8), 1197–1213. https://doi.org/10.1007/s10530-007-9196-8

Reitzel, A. M., Sullivan, J. C., & Finnerty, J. R. (2010). Discovering SNPs in protein coding regions with stellasnp: Illustrating the characterization and geographic distribution of polymorphisms in the estuarine anemone nematostella vectensis. *Estuaries and Coasts*, *33*(4), 930–943. https://doi.org/10.1007/s12237-009-9231-3

Tarrant, A. M., Helm, R. R., Levy, O., & Rivera, H. E. (2019). Environmental entrainment demonstrates natural circadian rhythmicity in the cnidarian Nematostella vectensis. *Journal of Experimental Biology*, *222*(21). https://doi.org/10.1242/jeb.205393

Voolstra, C. R., & Ziegler, M. (2020). Adapting with Microbial Help Microbiome Flexibility Facilitates Rapid Responses to Environmental Change. *BioEssays*, *42*(7), 2000004. https://doi.org/10.1002/bies.202000004
